# Candidate Urine Peptide Biomarkers for IgA Nephropathy: Where Are We Now?

**DOI:** 10.1155/2018/5205831

**Published:** 2018-01-21

**Authors:** Karolina Marek-Bukowiec, Andrzej Konieczny, Krzysztof Ratajczyk, Wojciech Witkiewicz

**Affiliations:** Regional Specialist Hospital in Wrocław, Research and Development Center, Wrocław, Poland

## Abstract

Early detection, prognosis, and management of IgA nephropathy (IgAN) remain a challenge. Histological examination of renal tissue still comprises the only way to confirm an IgAN diagnosis. It is of great importance to establish noninvasive diagnostic, prognostic, and predictive biomarkers that would improve the clinical care and outcome of patients suffering from IgAN. This review summarises the findings from previous mass spectrometry- (MS-) based studies dedicated to the discovery of urinary peptide profiles specific to IgAN. There is a substantial number of urinary peptides that have been discovered to date, which show promise as biomarkers of IgAN; however, all of them require further, rigorous validation in well-planned studies, involving a large number of subjects who represent diverse and numerous populations.

## 1. Introduction

IgA nephropathy (IgAN, Berger's disease) is one of the most predominant variants of primary glomerular disease, ultimately leading to end-stage renal disease (ESRD) in a great proportion of patients (approximately 30–40%) [1]. Renal biopsy remains the only tool providing a definitive diagnosis of IgAN and the only aid in making optimal therapeutic decisions [2]. However, kidney biopsy carries a considerable risk of potential complications like pain, fever, perirenal hematoma, or hematuria requiring blood transfusion or surgical intervention and is not always feasible due to coexisting conditions (e.g., uncontrolled hypertension, blood coagulation anomalies, anatomic abnormalities, and pregnancy) or the lack of patient consent [3–5]. Thus, there is an urgent need to develop noninvasive biomarkers which are able to provide reliable diagnostic, prognostic, and predictive information in IgAN that would supplement or preferably outperform renal biopsy.

Urinary peptidomics is an emerging and promising field for clinical biomarker discovery in IgA nephropathy. Many previous studies have shown that the urinary peptide profiles of IgAN patients differ significantly both from those suffering from other chronic renal diseases and from other healthy controls [6–8]. Most of the urinary, endogenous peptides stem from the kidneys and the urinary tract and possess a specific expression profile, shaped by physiological and pathophysiological processes ongoing in the urinary system. Thus, a quantitative and qualitative examination of the urinary peptidome of IgA nephropathy patients may facilitate the discovery of specific biomarkers, and in parallel, help to uncover molecular mechanisms driving IgAN. Developing a reliable, clinically useful, urine peptide biomarker/biomarker panel, with the use of mass spectrometry, is not trivial, mostly due to the strong interlaboratory variation in experimental design (e.g., size and composition of cohorts), sample collection (e.g., first versus second morning urine), sample processing (different strategies to isolate urine peptides), and data analysis (various MS platforms and data analysis tools). All of this leads to incomparable datasets and an inability to conduct a meta-analysis to validate the candidate biomarkers [9]. Biomarker discovery in proteomics is also hampered by the complexity of urine samples, a wide range of protein concentration, peptide normalisation difficulties, and the confounding effect of numerous variables (e.g., urine pH, age, and diet), influencing the stability and composition of urinary peptide patterns and contributing to false-positive findings [10–12].

In this review, we summarise the current state of the literature regarding urinary peptide profile “specific” for IgA nephropathy, originating from previous mass spectrometry-based studies. We also discuss some of the critical variables that can markedly influence the peptide profile of urinary specimens and confound data interpretation.

## 2. Materials and Methods

In order to summarise the knowledge regarding putative urinary peptide biomarkers of IgA nephropathy identified by mass spectrometry, we have searched the electronic bibliographic databases PubMed and Google Scholar, using advanced search options. The search was performed using various combinations of the following keywords: “IgAN,” “IgA nephropathy,” “chronic kidney disease,” “CKD,” “urinary peptidome,” “urinary peptides,” “urinary proteome,” “urinary peptide biomarkers,” “urinary peptide pattern,” “urinary peptide profile,” “proteomic analysis of urine,” “urine proteomics,” and “proteomic study.” We limited our search to original publications written in English, published between 1990 and 2017, including the search terms in the title or in the abstract. The exclusion criteria for the articles were as follows: no full PDF available, review article, letter, comment, case report, or conference abstract. In total, seven original articles were found that met the inclusion criteria.

## 3. IgA Nephropathy

IgA nephropathy is a glomerular disease that can be recognised only by histopathological examination of the renal biopsy specimen, which reveals the presence of dominant or codominant mesangial deposits of IgA immunoglobulin [13]. The disease can be diagnosed as primary if it is confined only to the kidney or secondary if it comprises a renal manifestation of a systemic disease, like chronic liver disease, diabetes, hypertension, amyloidosis, or lupus [14]. IgAN remains the dominant form of primary glomerulopathies in adults, with a global prevalence of 2.5 cases per 100,000 per year [15]. According to registries of glomerular diseases, IgAN incidence ranges from 5% in the Middle East [16] to 10–35% in Europe [17] and up to 50% in China or Japan [18]. IgAN incidence is probably strongly underestimated, as not every patient with suspected kidney disease undergoes renal biopsy. The striking data from necropsy studies revealed that the prevalence of IgA deposits in the general population may range from 2.4% to even 16% [19–21]. The clinical course and outcome of primary IgAN are strongly variable and unpredictable. The symptoms may range from microscopic hematuria (benign condition, usually asymptomatic) to subnephrotic proteinuria to nephrotic proteinuria with gross hematuria (advanced, symptomatic) [22]. Nephrotic range proteinuria, hypertension, decreased estimated glomerular filtration rate (eGFR), and histological grading are robust predictors of adverse renal outcome in IgA nephropathy [23]. In most cases, the disease progresses over a long period of time and eventually leads to end-stage renal disease (ESRD) in a large proportion of patients (even 50% within 20 years) [24]. The diagnosis of primary glomerular diseases is challenging and highly complex. It always requires a kidney biopsy, an invasive clinical procedure, associated with a substantial risk (around 3%) of complications like severe pain, infections, or serious bleeding [25–30]. Additionally, renal biopsy always requires hospital admission and sometimes cannot be performed due to coexisting systemic diseases (e.g., uncontrolled hypertension, atherosclerosis, diabetes mellitus, amyloidosis, and blood coagulation abnormalities) or the lack of patient consent [30]. Other disadvantages include the high cost of the procedure and the time-consuming protocol of sample processing and analysis [31, 32].

Future studies should put great effort into discovering noninvasive, specific biomarkers of primary IgAN that will permit an early detection as well as reliable monitoring and prediction of its course.

## 4. Urinary Peptidome

The peptidome is generally considered to be a fraction of low-molecular weight (LMW) proteome, encompassing amino acid oligomers and polymers, with a molecular mass below 30 kDa, that does not require complex processing (e.g., trypsin digestion), prior to mass spectrometry analysis [33–35]. Peptides act as central keepers of homeostasis, affecting and integrating the nervous (neurotransmitters, neuromodulators), endocrine (peptide hormones), and immune system (antimicrobial peptides) [36]. These molecules are mostly generated by the proteolytic breakdown of larger precursor proteins, remaining inactive until cleaved (e.g., the conversion of proinsulin into insulin) [37, 38]. Due to the significantly varying activity of proteases in physiological and pathologic states, the proteolytic peptide pattern may be used to determine the activity of proteases in the context of a specific condition [38]. Recent computational and ribosome profiling studies have revealed that the peptidome also contains numerous peptides, directly translated from short open reading frames (mRNA, lncRNA) [39–41]. The short open reading frame- (sORF-) encoded peptides remain functionally uncharacterised, with the exception of humanin, encoded in the mitochondrial genome (75 bp ORF), and possessing proved neuroprotective and cytoprotective properties [42].

The peptides produced both by normal and diseased body tissues are widely distributed throughout the body fluids, for example, blood, urine, cerebrospinal fluid, and saliva, and comprise an attractive reservoir for biomarker discovery [38]. Urinary proteome/peptidome is of special interest as urine is a noninvasively accessible body fluid that can be obtained repetitively in “large” amounts and most importantly, it is characterised by low proteolytic activity (e.g., in comparison to blood). Urine samples can be stored for hours at room temperature or kept at 4°C, without significant alterations in the proteomic pattern [43]. This is probably due to the fact that proteolytic degradation has already occurred while the urine was “stored” in the bladder [34]. However, if urine is collected for proteomic analysis, particular attention should be paid to the urinary pH, which may significantly contribute to the changes in the urinary peptidomic profile. Previous studies have revealed that a close to neutral urine pH “assures” protein stability (e.g., due to the activity of trypsin and inter-alpha-trypsin inhibitors), whereas an acidic pH (<6.0) shifts toward the activation of acid endoproteases (e.g., cathepsin D, aspartic protease) and the generation of “artificial” peptides [12, 44, 45]. The human urine pH ranges from strongly acidic (4.5) to alkaline (8.0) and is remarkably influenced by diet, medications, and underlying diseases [10]. Thus, urine pH should be determined immediately after collection and adjusted to neutral, if necessary, to ensure reliable and repeatable results. Apart from a fairly stable peptidomic pattern, another advantage of the urine as a “biomarkers mine” is the “low” complexity and “low” dynamic range of proteins/peptides expression, in comparison to plasma, that is 10^6^ for urine and 10^9^ for plasma [46, 47]. Normal human urine contains at least 5000 naturally occurring peptides (<20 kDa), originating from glomerular filtration, tubular secretion, and epithelial cells, which line the kidneys and urinary tract [48, 49]. As much as 70% of the low-molecular weight proteins come directly from the renal system [50], it is estimated that approximately 49% of the total urinary proteins/peptides remain associated with the urine supernatant, 48% belongs to the debris and cell fraction, and around 3% remains entrapped in the exosomes [50, 51]. The urinary fraction should be considered as a separate source of potential peptide biomarkers, as they possess diverse protein/peptide composition and provide various types of biological information [51]. The urinary peptidome faithfully reflects the physiological and pathophysiological processes, ongoing in the urinary system, as well as in other parts of the body. Thus, it is widely utilised as a “gold mine” for biomarker discovery for both the urinary system, as well as for nonurinary system-related diseases [7, 52–54].

Urinary peptidomics is a powerful tool, although not free of challenges. Some of the major problems include contamination with organic and inorganic urinary components, persisting in the protein sample even after extensive cleanup and interfering with the mass spectrometry analysis. Another obstacle are the intra- and interindividual differences in protein/peptide concentration requiring complex normalisation strategy. Finally, the exogenous and endogenous variables (e.g., age, smoking, diet, exercise, and environmental factors) significantly alter the proteomic/peptidomic pattern and hamper the urinary biomarker research [6, 55].

One of the previous studies showed that coffee consumption is associated with changes in the expression of 11 urinary proteins, including metalloproteinase inhibitor 2 that was earlier considered as a putative biomarker of bladder cancer [56]. Another example of a confounding variable is age. As shown by Zürbig et al., the urinary peptide profile changes significantly with aging; thus, it is critically important to pay attention to the age distribution of discovery and validation cohorts in the course of biomarker discovery projects [48]. In a previous study, by Haubitz et al., the IgAN cohort included 11% of patients aged above 60 years, thus raising the question of the potential confounding effect of age on the results [6].

Urinary peptidome is certainly an ideal site to search for biomarkers of human diseases, including IgA nephropathy; however, urine proteomics studies have to be planned in detail and critically, by taking into consideration all the potential confounding variables that may influence the accuracy and reliability of the results.

## 5. Urinary Peptide Profiles of IgA Nephropathy

The study published in 2005, by Haubitz et al., provided the first evidence of urinary protein/peptide profile utility in discrimination between IgAN patients, healthy individuals, and membranous nephropathy (MN) subjects and its potential application as a diagnostic tool (Table 1) [6]. The authors also noticed that the IgAN-related profile changes significantly in patients receiving an increasing number of antihypertensive drugs, which implies that urinary peptidome profiling may theoretically serve as a valuable means for evaluating treatment efficacy and testing of novel, promising therapies [6]. The diagnostic potential of the urinary peptidome in IgA nephropathy was further confirmed by Julian et al., who developed a 25-peptide panel, distinctly separating IgAN subjects from healthy controls and from patients with other renal diseases (e.g., FSGS, diabetic nephropathy, and amyloidosis) with an overall specificity of 82.3% (Table 1) [7]. Unfortunately, the signatures proposed both by Haubitz and Julian have been developed based on relatively small cohorts (specifically IgAN), not properly matched regarding age, using unfractionated urine (Table 1). The analysis of peptides derived from urine supernatant, performed by Graterol et al., resulted in the identification of 16 low-molecular weight proteins, discriminating between IgAN patients and healthy controls. The signature included uromodulin (UMOD), alpha-1-antitrypsin (A1AT) peptides, and beta-2-microglobulin (B2M) fragment. The level of uromodulin (m/z 1898) and alpha-1-antitrypsin peptide (m/z 1945) was found to correlate negatively and positively, respectively, with the lesion severity in IgAN (Table 1). The same group defined a 10-peptide multimarker, composed of UMOD peptides, A1AT fragment, and low-molecular weight proteins, with an unknown amino acid composition that strongly correlated with the doubling of serum creatinine in IgAN patients (Table 1) [57]. However, it should be stressed that the aforementioned study lacked the CKD control group and thus did not evaluate whether the “putative peptide markers” are truly specific for IgA nephropathy. There is currently a high demand for noninvasive biomarkers, possessing the ability to accurately discriminate between different variants of chronic kidney diseases (correlating with histopathological results of renal biopsy), which would enable the monitoring of their clinical course. Another study has revealed that a set of 11 peptides found in urine supernatant possesses diagnostic potential in IgAN. The UMOD (m/z 1913.14) fragment, that was the only well-characterised component of the signature, was underrepresented in the urine samples of IgAN subjects in comparison to healthy individuals (Table 1) [58]. Uromodulin is a kidney-specific molecule and the most abundant protein in normal human urine, playing an immunosuppressive role [59]. As the diminished expression of particular UMOD fragments accompanies a number of glomerulopathies, the UMOD level alone cannot be considered as a specific biomarker of IgAN. Another proteomic study found that A1AT protein isoforms and their cleavage products are significantly elevated in urine and renal cortex samples of IgAN patients, in comparison to the healthy control group (Table 1) [60]. A1AT is the main blood serine proteinase inhibitor, possessing a broad spectrum of inhibitory activities and exerting anti-inflammatory effects; thus, its degradation might potentially play a role in IgAN pathogenesis [60]. Unfortunately, the study did not assess the diagnostic value of A1AT, in relation to various types of chronic kidney diseases.

Urinary laminin G-like 3 (m/z 21,598) and free K light chains (m/z 23,458) are other candidates for peptide biomarkers of IgA nephropathy that were shown to possess the potential to differentiate between IgAN patients, subjects with other chronic kidney diseases, and healthy controls (Table 1). The expression of these peptides was found to correlate inversely with the severity of clinical and histologic features of IgAN, as well as with the clinical outcome [61].

The majority of the previous MS-based peptidomic studies which focused on finding urine peptide biomarkers for IgAN, suffered from insufficient sample sizes and the lack of a truly heterogeneous control group (wide spectrum of renal diseases). The studies conducted so far employed distinct strategies for protein sample preparation and analysis which explains the very small overlap between the “IgAN-specific peptide patterns” originating from distinct studies.

The meta-analysis of proteomic data conducted recently by Siwy et al. represents the first attempt to identify peptide signatures specific for IgAN, by utilising impressively large cohorts. The combined, retrospective analysis of 1180 independent CKD proteomic data sets, yielded a 116-peptide signature, clearly discriminating IgAN condition (179 cases) from other common variants of chronic kidney diseases (*n* = 1001) (Table 1) [8]. The IgAN peptide classifier extracted from merged datasets included a number of protein fragments, recently proposed as potential biomarkers of IgAN [6, 7]. 12% of peptides identified previously by Julian et al. overlapped with the 116 multimarkers, and these were mostly collagen fragments [7]. The diagnostic potential of the proposed signature requires consequent validation in large, multicentre prospective studies that will involve IgAN patients and a broad spectrum of CKD cases.

In a recent study, Good et al. developed a 273 urinary peptide signature (CKD273-classifier), accurately discriminating patients with CKD (independently on aetiology) from healthy and disease controls. The study included an extremely large cohort of 3600 urine samples (including IgAN), collected at several dozen clinical centres in Europe, America, and Australia and processed according to the same standard protocol to assure consistency [43]. This is the first diagnostic peptide signature for CKD, positively validated in prospective studies, which has received a letter of approval from the FDA [62].

## 6. Conclusions

IgA nephropathy is the predominant subtype of primary glomerular disease and one of the most common causes of chronic kidney disease (CKD) worldwide in adults. The current diagnosis of IgAN is based entirely on the evaluation of tissue specimens obtained by kidney biopsy, an invasive diagnostic procedure, carrying a considerable risk of minor and major complications [2, 63]. To date, there are no molecular strategies available that might serve as a noninvasive alternative to the renal biopsy (so-called liquid biopsy). Therefore, there is an urgent need to develop specific and sensitive biomarkers that may be utilised for screening, diagnosis, and prognosis, as well as monitoring of IgAN.

With the advances in mass spectrometry-based proteomics technology in the last decade, it has become possible to globally analyse the protein/peptide composition of various cell types, tissues, and body fluids, for example, blood, serum, urine, amniotic fluid, or cerebrospinal fluid [64]. Urine comprises an excellent biological material for proteomic research, as it can be obtained in large volumes, in a fully noninvasive manner and possesses stable proteome [65]. The urinary peptidome represents the low-molecular weight fraction of the urinary proteome and comprises a valuable source of kidney disease-specific biomarkers, as its pattern changes markedly during the course of the disease [6, 7, 9].

There are many potential urine peptide biomarkers for IgAN identified to date; however, all of them require further rigorous validation. More studies are mandatory to uncover the qualitative and quantitative profiles of urinary peptides that are related specifically to IgA nephropathy. Future studies should employ large cohorts of IgAN subjects and patients with other chronic kidney diseases, who will represent various and numerous populations. It would be crucial to develop and follow standardised procedures for urine peptidomics (i.e., sample collection, storage, and sample preparation for analysis and quality control), thus ensuring accurate MS-data reproduction by different laboratories. The candidate peptide biomarkers should undergo an extensive interlaboratory validation, to demonstrate their sufficient discriminatory power and suitability for clinical application.

## Figures and Tables

**Table 1:** Urine peptide signatures for IgA nephropathy identified previously by mass spectrometry-based studies.

						Independent validation								
			Sample size			Sample size			Discriminative ability of the biomarker					
Authors	Signature	Clinical utility	Primary IgAN	Healthy controls	Disease controls	Primary IgAN	Healthy controls	Disease controls	Sensitivity	Specificity	AUC (95% CI)	First versus second urine	Urine fraction	Proteomic strategy
Haubitz et al. [6]	22 proteins/peptides	Diagnostic, potentially predictive	45	57	13 (MN)				IgAN versus healthy controls (100%), IgAN versus disease controls (77%)	IgAN versus healthy controls (90%), IgAN versus disease controls (100%)		Second	Total urine	CE-MS

Julian et al. [52]	25 peptides	Diagnostic	45	207	253 (FSGS, DN, LN, N, amyloidosis, acute vasculitis with nephritis, hypertensive renal disease, MCD, membranous GN)	10	12	22 (HSP nephritis, non-IgA GN, IgA-positiveimmune-complexGN) and 5 HSP without nephritis		IgAN versus healthy and disease controls (82.3%)		Second	Total urine	CE-MS

Graterol et al. [57]	16 peptides	Diagnostic	19	14	No							First	Urine supernatant	MALDI-TOF MS
	10 peptides	Correlation with poor renal function												

Wu et al. [58]	11 peptides	Diagnostic	25	24	23 (MN, MCD, LN)	7	6	13 (MN, MCD, LN)	IgAN versus healthy controls (100%), IgAN versus disease controls (85.7%)	IgAN versus healthy controls (100%) IgAN versus disease controls (76.9%)		First	Urine supernatant	MALDI-TOF MS

Kwak et al. [60]	Alpha-1-antitrypsine isoforms and their fragments elevated in IgAN versus controls	Diagnostic	8	5	No							First	Urine supernatant	2-DE and MALDI-TOF-MS

Rocchetti et al. [61]	13 peptides including Perlecan laminin G-like 3 peptide and free K light chains	Diagnostic	49	40	42 (MGN, DN, N)	14		14 (MN, DN, N) and 10 MPGN				First	Urine supernatant	SELDI-TOF-MS

Siwy et al. [8]	116 peptides	Discriminating IgAN from dominant CKD	179	No	1001 (FSGS, MCD, MN, DN, N, LN)	57		417 (FSGS, DN, N, MCD, MN, LN, vasculitis-inducedkidney disease)			0.82 (0.76–0.87)	Second	Total urine	CE-MS

MN: membranous nephropathy; MCD: minimal change disease; LN: lupus nephritis; FSGS: focal segmental glomerulosclerosis; DN: diabetic nephropathy; N: hypertensive nephrosclerosis; MGN: membranous glomerular nephropathy; MPGN: non-IgA mesangioproliferative GN; HSP: Henoch–Schönlein purpura.
